# Metabolic Disturbances Associated with Beta-Blocker Therapy: New Insights from a Large-Scale Pharmacovigilance Study

**DOI:** 10.3390/ph19081150

**Published:** 2026-07-24

**Authors:** Crina Cristina Solomon, Carmen Maximiliana Dobrea, Anca Butuca, Adina Frum, Nastaca Alina Palade, Alina Liliana Pintea, Dragoș Anton Dădârlat, Steliana Ghibu, Ioana Rada Popa Ilie, Claudiu Morgovan, Mariana Cornelia Tilinca, Felicia Gabriela Gligor

**Affiliations:** 1 Preclinical Department, Faculty of Medicine, “Lucian Blaga” University of Sibiu, 550169 Sibiu, Romania; crinacristina.solomon@ulbsibiu.ro (C.C.S.); anca.butuca@ulbsibiu.ro (A.B.); adina.frum@ulbsibiu.ro (A.F.); nastacaalina.coman@ulbsibiu.ro (N.A.P.); claudiu.morgovan@ulbsibiu.ro (C.M.); felicia.gligor@ulbsibiu.ro (F.G.G.); 2 County Emergency Clinical Hospital Sibiu, 550025 Sibiu, Romania; alina-liliana.pintea@ulbsibiu.ro; 3 Department of Dental Medicine and Nursing, Faculty of Medicine, “Lucian Blaga” University of Sibiu, 550169 Sibiu, Romania; dragos.dadarlat@ulbsibiu.ro; 4 Department of Pharmacology, Physiology and Pathophysiology, Faculty of Pharmacy, “Iuliu Haţieganu” University of Medicine and Pharmacy, 6A Louis Pasteur Street, 400349 Cluj-Napoca, Romania; steliana.ghibu@umfcluj.ro; 5 Department of Endocrinology, Faculty of Medicine, “Iuliu Hațieganu” University of Medicine and Pharmacy, 3-5 Louis Pasteur Street, 400349 Cluj-Napoca, Romania; ioana.ilie@umfcluj.ro; 6 Department of Internal Medicine I, Faculty of Medicine in English, George Emil Palade University of Medicine, Pharmacy, Science, and Technology of Târgu-Mures, 540142 Târgu Mureș, Romania; mariana.tilinca@umfst.ro; 7 Clinical Compartment of Diabetes Mellitus, Nutrition and Metabolic Diseases, Emergency Clinical County Hospital of Targu Mures, 540136 Târgu Mureș, Romania

**Keywords:** beta-blockers, metoprolol, carvedilol, bisoprolol, nebivolol, pharmacovigilance, EudraVigilance, metabolic disturbances, dysglycemia, type 2 diabetes mellitus, obesity, disproportionality analysis

## Abstract

**Background**: Beta-blockers (BBs) remain a foundation of cardiovascular therapy. However, cumulative evidence suggests that metabolic adverse effects may differ significantly between individual sub-classes. While conventional BBs have been associated with changes in glucose and lipid metabolism, the comparative real-world reporting patterns of these events have not been comprehensively evaluated. **Objective**: We investigated the association between selected BBs and metabolic diseases using a large pharmacovigilance database and compared the reporting profiles of metoprolol (MET), carvedilol (CAR), bisoprolol (BIS), and nebivolol (NEB). **Methods**: A descriptive and disproportionality analysis was performed using Individual Case Safety Reports (ICSRs) submitted to the EudraVigilance database up to 26 April 2026. Metabolic adverse events were grouped into pathophysiological categories, including dysglycemia and type 2 diabetes mellitus (T2DM), lipid disorders, obesity-related disorders, inflammatory biomarkers, and hepatic steatosis. Reporting odds ratios (RORs) with 95% confidence intervals (CI) were calculated to identify disproportional reporting signals. To improve clinical reliability, a stratified analysis restricted to healthcare professional (HP)-submitted reports was conducted. **Results**: A total of 63.744 ICSRs were identified for the four BBs, including 27.169 for MET, 21.860 for BIS, 9487 for CAR, and 5228 for NEB. Dysglycemic events represented the most frequently reported metabolic category, with hyperglycemia and T2DM accounting for 155 reports each. MET was associated with the highest number of reports for T2DM, obesity, hepatic steatosis, and elevated triglycerides. In HP-submitted reports, MET showed significantly higher reporting odds of increased triglycerides, obesity, inflammation, and T2DM than BIS and/or CAR. CAR showed higher reporting odds for hyperglycemia and obesity compared with BIS, whereas BIS and, particularly, NEB presented comparatively lower reporting frequencies for metabolic diseases. No significant differences were observed for hepatic steatosis, decreased high density lipoprotein cholesterol (HDL-C), or impaired glucose tolerance. **Conclusions**: This large-scale pharmacovigilance study highlights substantial heterogeneity in the reporting of metabolic adverse events among commonly prescribed BBs. MET and to a lesser extent CAR were associated with higher reporting odds of metabolic diseases, whereas BIS and especially NEB demonstrated more favorable reporting profiles. Although causality cannot be established from spontaneous reporting data, these findings support individualized BB selection and closer metabolic monitoring in patients with cardiometabolic risk factors.

## 1. Introduction

Beta-adrenergic receptor antagonists (beta-blockers (BBs)) remain among the most widely prescribed cardiovascular medications worldwide and continue to represent a base of therapy for cardiovascular disorders [1]. Despite the introduction of newer therapeutic classes, BBs retain a central role in contemporary cardiovascular management due to their proven benefits in reducing morbidity and mortality in selected patient populations [2], which has led to their incorporation into international guideline recommendation for ischemic heart disease, chronic heart failure, arrythmias, and several other cardiovascular conditions. Consequently, millions of patients are exposed to BB therapy for prolonged periods, highlighting the importance of a comprehensive understanding of their long-term safety profile [3]. 

Although BBs are generally regarded as a single therapeutic class, substantial heterogeneity exists among different agents with respect to receptor selectivity, additional pharmacological properties, and metabolic effects [4]. Traditionally, BBs are classified according to their relative affinity for β1- and β2-adrenergic receptors. Cardioselective agents, such as atenolol, bisoprolol (BIS), metoprolol (MET), and nebivolol (NEB), preferentially block β1-adrenergic receptors located predominantly in the myocardium, kidneys and adipocytes, whereas β2-adrenergic receptors are expressed mostly in the vasculature and smooth muscles of the airways, as well as in the heart and a variety of other cell types. Activation of β2-adrenergic receptors also increases energy expenditure and improves glucose homeostasis [5]. On the other hand, the blockade of β2-adrenoreceptors causes vasoconstriction, bronchoconstriction and disturbances of metabolic function [6].

Activation of β3-adrenoreceptors increase the production of nitric oxide (NO), which reduces cardiac contractility and induces vasodilatation in the peripheral arteries [7]. In addition, they can activate brown adipose tissue as well as stimulate the burning of white adipose tissue, with associated increased thermogenesis and energy expenditure [8].

Recent reviews have highlighted the complex interaction between BBs and metabolic pathways, demonstrating that the metabolic impacts of these agents extend beyond simple adrenergic receptor blockades and may involve the modulation of inflammatory processes, oxidative stress, endothelial function, and NO-mediated signaling [9]. The third generation of BBs, such as carvedilol (CAR) and nebivolol, have been shown to exert more favorable effects on insulin sensitivity, endothelial function, and metabolic homeostasis compared with conventional BBs [10,11]. Therefore, new evidence suggests that the metabolic consequences of BBs should not be considered a uniform class effect but rather the result of complex pharmacodynamic differences among intra-class drugs [12]. The recognition of this pharmacological heterogeneity is particularly relevant when evaluating adverse metabolic events, as differences in receptor selectivity and additional mechanisms may influence the risk of dysglycemia, weight gain, lipid abnormalities, and other metabolic disturbances observed during long-term treatment [13,14,15]. These effects are of clinical relevance given the growing global burden of obesity, metabolic syndrome (MS), and type 2 diabetes mellitus (T2DM), conditions that frequently coexist with cardiovascular diseases [16]. Among the reported metabolic consequences, dysglycemia has received considerable attention [17]. Several studies have shown that commonly non-vasodilating BBs, mainly atenolol and metoprolol, have been associated with impaired insulin resistance, worsening glycemic control, and an increased risk of new-onset diabetes, particularly in people with underlying cardiometabolic risk factors [18]. Proposed mechanisms include decreased insulin-mediated skeletal muscle perfusion, the inhibition of pancreatic insulin secretion, impaired glucose uptake, and alterations in the sympathetic regulation of carbohydrate metabolism [19]. In contrast, third-generation vasodilatory BBs such as carvedilol and nebivolol appear to exert more favorable effects on glucose metabolism, potentially through improvements in endothelial function, NO bioavailability, and peripheral insulin sensitivity [20]. These observations suggest that metabolic safety may vary substantially across BB subclasses rather than representing a uniform class effect [21].

In addition, some BBs have been linked to modest weight gain through different pathways [22]. Reduced thermogenesis, diminished exercise tolerance, lower resting energy expenditure, and alterations in substrate utilization have been proposed as mechanisms contributing to these adverse effect [23]. Even modest weight gain may have significant implications in patients with obesity or MS, potentially exacerbating insulin resistance and increasing the risk of diabetes and other metabolic complications [16]. Given the bidirectional relationship between adiposity and cardiovascular disease, understanding drug-related effects on body weight is increasingly relevant in long-term cardiovascular pharmacotherapy [24].

On the other hand, some BBs have been associated with adverse effects on lipid metabolism, including increased triglyceride concentrations and decreased high-density lipoprotein cholesterol (HDL-C) levels [25]. Collectively, these findings suggest that metabolic disturbances represent an important but incompletely characterized dimension of BB safety [26].

However, accumulating evidence indicates significant pharmacological heterogeneity within the class with respect to receptor selectivity, intrinsic sympathomimetic activity, membrane-stabilizing effects, and vasodilatory properties [27]. This growing recognition that BBs differ considerably in their metabolic properties has challenged the historical perception of a homogeneous drug class and has important implications for individualized treatment selection [28]. Although randomized clinical trials and observational studies have provided valuable insights into BB-associated metabolic disturbances, important knowledge gaps remain [29]. Randomized clinical trials often focus primarily on cardiovascular efficacy and might not be sensitive enough to capture uncommon or delayed metabolic adverse events [30]. Observational studies are frequently limited by relatively small sample sizes, restricted patient populations, short follow-up durations, or a primary focus on efficacy outcomes rather than adverse metabolic events. Furthermore, uncommon, delayed, or unexpected metabolic reactions may be underpowered during pre-marketing development or conventional clinical research. Consequently, the full spectrum of metabolic adverse events associated with BBs in routine clinical practice remains incompletely understood [31].

Pharmacovigilance databases provide a valuable complementary approach for evaluating drug safety in real-world settings. By collecting spontaneous adverse event reports from diverse populations and healthcare systems, these databases can identify both established and emerging safety concerns, complementing evidence derived from clinical trials and observational research [32]. Given the widespread and chronic use of BBs, large-scale pharmacovigilance analyses may offer important insights into their metabolic safety signal and facilitate more personalized therapeutic decision-making [33].

Therefore, this present study aimed to comprehensively investigate the reporting association between BB therapy and metabolic disturbances (including dyslipidemia, hyperglycemia and T2DM, which are often inconsistently documented in the clinical literature) using a large pharmacovigilance database. Although dyslipidemia, hyperglycemia and T2DM are recognized as adverse metabolic outcomes associated with certain BBs, they have been evaluated inconsistently across clinical studies. Differences in study design, patient populations, BB subclasses, outcome definitions, follow-up duration and the primary focus of cardiovascular trials have resulted in heterogenous and sometimes conflicting evidence regarding their incidence and comparative risk. The therapeutical agents selected for this study were metoprolol (MET), bisoprolol (BIS), carvedilol (CAR), and nebivolol (NEB), as these have been identified among the most frequently prescribed BBs in the literature [34,35,36]. By performing a disproportionality analysis exclusively on reports submitted by healthcare professionals (HPs) within the EudraVigilance database, we aim to provide a clinically informed assessment of the reporting patterns associated with MET, BIS, CAR, and NEB, thereby enabling a comparative evaluation of their reporting profiles. To our knowledge, no previous pharmacovigilance study has comprehensively compared the reporting patterns of a broad range of metabolic ADRs across individual BBs using the EudraVigilance database. Addressing this gap, the present study may provide complementary real-world evidence regarding potential differences in the metabolic safety profiles of commonly prescribed BBs.

## 2. Results

### 2.1. Descriptive Analysis

**Figure 1:**
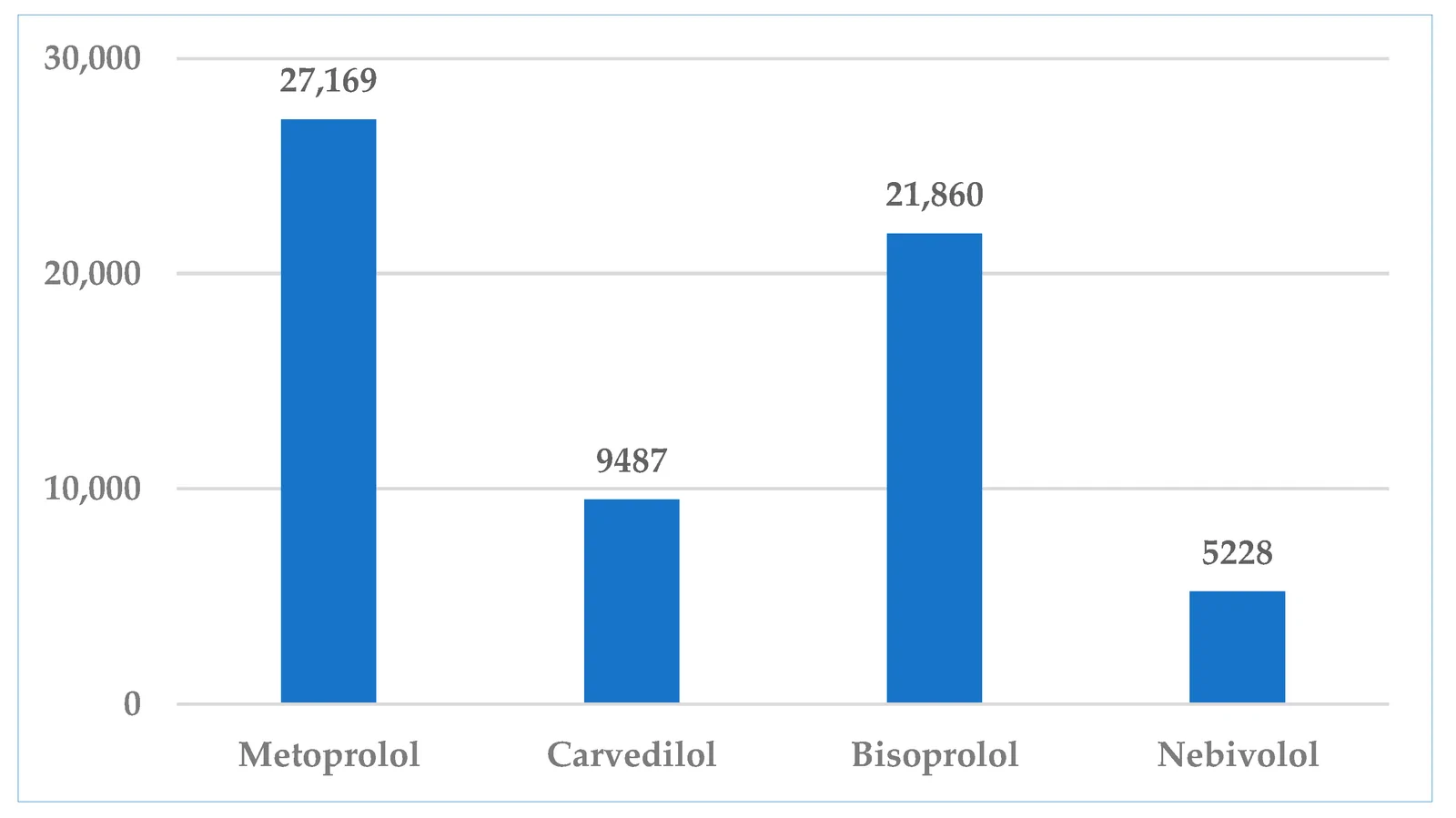
Distribution of cases reported in the EudraVigilance database until 26 April 2026.

According to Figure 1, the highest number of cases reported in the EV database was for MET (*n* = 27,169) and the lowest was for NEB (*n* = 5228). Also, a high number of cases were registered for BIS (*n* = 21,860).

The general characteristics are presented in Table 1. Regarding the distribution of cases by age, the most frequent cases have been reported in adult and elderly populations. Patients aged 65–85 years accounted for all BBs (CAR: 41.3%, BIS: 43.5% and NEB: 40.6%), except for MET, which had the highest frequency in the 18–64 year-old group (38.0%). Reports concerning patients older than 85 years were most frequently observed with BIS (12.2%), followed by NEB (8.4%), CAR (8.0%) and MET (6.6%). Pediatric reports (<18 years) represented a small proportion of cases for all agents, each accounting for less than 1% within individual age categories.

On the other hand, female patients were more likely to report ADRs across all BBs (MET: 53.7%, BIS: 52.5%, and NEB: 54.9%), whereas reports with associated CAR were more frequently reported in males (53.2%). The proportion of reports with missing sex information was low, ranging from 2.1% to 4.8%.

Geographical distribution revealed substantial differences among the studied BBs. Most reports involving NEB (80.3%) and BIS (77.9%) originated from EEA countries, whereas MET (57.2%) and CAR (66.90%) reports were most frequently from non-EEA countries.

However, most cases have been reported by HPs across all four BBs, accounting for 69.30% of MET reports, 83.70% of CAR reports, 74.90% of BIS reports, and 74.20% of NEB reports. Reports submitted by non-HPs represented approximately one quarter for MET (30.2%) and the lowest for CAR (15.4%). Missing reporter information was uncommon across all groups.

Overall, the reporting populations were predominantly composed of adults and elderly patients, with HPs serving as the principal source of reports. Notable differences were observed in the geographic origin of reports and, to a lesser extent, in sex distribution among individual BBs.

The distribution of ADRs reported across MedDRA System Organ Classes showed broadly similar reporting patterns among the four BBs: (i) cardiac disorders were among the most commonly reported events, ranking first for CAR (32.0%) and BIS (28.8%), second for MET (30.1%), and third for NEB (21.2%); (ii) general disorders and administration site conditions represented the most frequently reported SOCs for MET (32.6%) and NEB (28.5%) and the second most frequently reported SOCs for CAR (31.2%) and BIS (27.4%); (iii) nervous system disorders consistently ranked among the three most frequently reported SOCs across all agents, accounting for MET: 26.1%, CAR: 23.5%, BIS: 23.8%, and NEB: 23.9% (Table 2).

### 2.2. Signal Detection at the Preferred Terms (PTs) Level

#### 2.2.1. Analysis of the Selected PTs

According to the data presented in Table 3, among the selected metabolic-related adverse events, diseases of glucose metabolism represented the most frequently reported pathophysiological category. Hyperglycemia and T2DM were the most reported PTs, each accounting for 155 reports across the four BBs. Hyperglycemia was most frequently reported with MET (*n* = 73) and CAR (*n* = 49), whereas reports of T2DM were predominantly associated with MET (*n* = 99), followed by BIS (*n* = 28) and CAR (*n* = 22). Additional glucose-related events included impaired glucose tolerance (*n* = 29), impaired fasting glucose (*n* = 4), and insulin resistance (*n* = 4), further supporting the prominence of dysglycemic events within the reporting database.

Inflammation-related adverse events also represented a notable category. Increased C-reactive protein (CRP) was the most frequently reported inflammatory marker (*n* = 118), with the highest number of reports observed for BIS (*n* = 62). Reports coded as inflammation (*n* = 110) were most associated with MET (*n* = 58) and BIS (*n* = 38).

Lipid abnormalities represented another important category of metabolic disturbances. An increased level of blood triglycerides (TGs) was the most reported lipid-related event (*n* = 103), occurring predominantly with MET (*n* = 64) and BIS (*n* = 28). Decreased high-density lipoprotein cholesterol (HDL-C) was reported less frequently (*n* = 36), although MET again accounted for the largest part of cases (*n* = 21).

Obesity-related adverse events were also identified. Obesity was reported in 63 cases overall, with the majority associated with MET (*n* = 37). Increased waist circumference (*n* = 13) and central obesity (*n* = 3) were less commonly reported. MS itself was reported in 13 cases, most frequently with MET (*n* = 8).

Hepatic manifestations potentially linked to metabolic dysfunction were represented by hepatic steatosis, which accounted for 70 reports overall. MET was associated with the highest cases reported (*n* = 36), followed by BIS (*n* = 17), CAR (*n* = 9) and NEB (*n* = 8).

#### 2.2.2. Disproportionality Analysis

**Figure 2:**
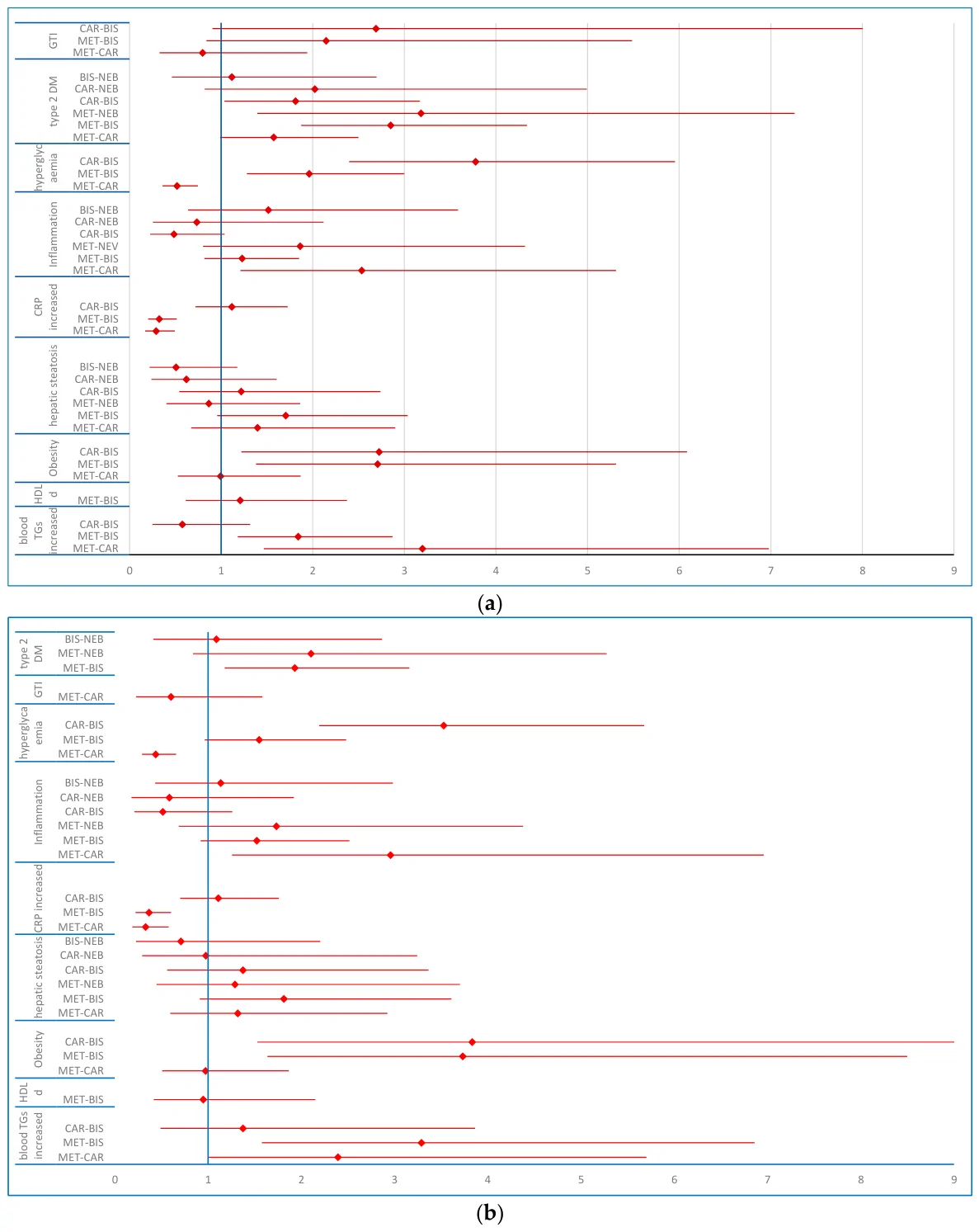
Disproportionality analysis in the BB group. (**a**) Overall dataset; (**b**) healthcare professional category.

Similar results were obtained from a disproportionality analysis of the overall dataset and from stratified analyses by the HP reporter category (Figure 2a,b). Some PTs were reported to have a higher probability of MET than the other BBs. Thus, in the HP group, “blood triglycerides increased” was associated as being more likely for MET compared to CAR (ROR: 2.39, 95%CI: 1.00–5.70) and BIS (ROR: 3.29, 95%CI: 1.58–6.86), “obesity” was more likely in comparison to BIS (ROR: 3.73, 95%CI: 1.64–8.50), “inflammation” was more likely compared to CAR (ROR: 2.96, 95%CI: 1.26–6.96), and “T2DM” was more likely compared to BIS (ROR: 1.93, 95%CI: 1.18–3.16). On the other hand, “CRP-increased” was reported as having a lower probability for MET compared to CAR (ROR: 0.33, 95%CI: 0.19–0.58) and BIS (ROR: 0.37, 95%CI: 0.22–0.60), and “hyperglycemia” was less likely for MET than CAR (ROR: 0.44, 95%CI: 0.29–0.66). Additionally, “obesity” (ROR: 3.83, 95%CI: 1.53–9.61) and “hyperglycaemia” (ROR: 3.53, 95%CI: 2.19–5.68) were reported more frequently for CAR than for BIS. No differences were noticed for “high density lipoprotein decreased”, “hepatic steatosis”, and “glucose tolerance impaired” (Figure 2).

## 3. Discussion

BBs are among the most effective medications for improving cardiovascular outcomes and remain widely prescribed due to their proven benefits in reducing morbidity and mortality across several cardiovascular conditions [37]. This present study extends the existing evidence by providing a comprehensive real-world comparison of metabolic adverse event reporting across four commonly prescribed BBs. Rather than evaluating isolated metabolic outcomes, our analysis demonstrates heterogeneous reporting patterns among individual agents, suggesting that the reporting of metabolic ADRs may differ substantially within the BB classes.

The large number of reports identified in EV (*n* = 63,744), among which almost half are represented by MET (*n* = 27,169) and approximately one third by BIS (*n* = 21,860) (Figure 1), could partially be linked to a combination of factors. On one hand, there is the high frequency of MET prescription in several large incidence diseases, such as hypertension, cardiac insufficiency, ischemic heart disease, migraine, arrythmias [38,39,40], BIS in hypertension, ischemic heart disease, and heart failure [41]. On the other hand, MET has been approved since 1975, with BIS following a decade later, allowing for a greater exposure than the more recently marketed NEB and CAR, starting in the mid-1990s [42,43,44,45].

An analysis of EV data showed a higher frequency of reports for MET among adults aged 18–64 years, whereas reports for the other BBs were more common in the 65–85 year-old age group. This pattern may reflect differences in prescribing practices, as conventional BBs such as MET are no longer routinely recommended as first-line therapy for uncomplicated hypertension in older adults without compelling indications. In addition, younger adults may be more likely to report ADRs, particularly through electronic reporting systems [46,47,48,49]. According to these results, ADRs were reported more frequently in women receiving MET, NEB or BIS than in the male group. Kalibala et al. showed that women generally experience higher drug exposure and a higher frequency of ADRs across antihypertensive drug classes, with particularly more ADRs reported for CYP2D6-dependent BBs [50]. Regarding MET, Eugene et al. demonstrated that women have more than a twofold greater systemic exposure than men following administration of the same 100 mg oral dose. Although these pharmacokinetic differences were reported, no sex-specific dose adjustment recommendations have been implemented. This may contribute to the higher frequency of ADRs reported in women [51]. On the other hand, ADRs were reported more frequently in men receiving CAR than in those receiving MET, BIS or NEB. Probably, the preferential use of CAR in males with heart failure could be one cause of these reporting trends [52]. Moreover, due to the inhibition of α_1_-adrenergic receptors, CAR could be preferred in the treatment of benign prostatic hypertrophy in patients with heart failure [53].

The origin of reports was another aspect analyzed in the present study. Compared with BIS and NEB, a higher proportion of ICSRs for MET and CAR originated from non-EEA countries. These differences may reflect regional prescribing practices and greater utilization of these agents [54,55]. For example, MET and CAR accounted for more than 70% of BB prescriptions in the United States in 2024. However, because exposure-adjusted denominators were unavailable, these findings should be interpreted cautiously [56,57].

Similarly, differences in overall prescribing frequency among the evaluated beta-blockers may also have contributed to the observed reporting patterns and should be considered when interpreting the comparative findings. ADR reports for BBs in the EEA come predominantly from HPs. This phenomenon could offer superior accuracy to the EV database, as reports submitted by HPs include essential parameters such as the exact therapeutic indication, the correct dose, and the patient’s comorbidity history. The direct involvement of the physician ensures an objective validation of the symptoms and a high fidelity in establishing the causal link between BB administration and the occurrence of an adverse reaction [58,59,60].

Most reports belonged to the MedDRA SOCs “Cardiac disorders”, “General disorders and administration site conditions”, and “Nervous system disorders”, consistent with the known pharmacological profile of BBs and previous real-world studies [61,62].

MET, a lipophilic second-generation BB, accounted for the highest number of reports related to metabolic syndrome, central obesity, and altered glycemic profile. This observation is biologically plausible, as previous studies have suggested that metoprolol may reduce insulin sensitivity and inhibit lipoprotein lipase, with clinical evidence also showing increased oxidative stress and impaired insulin sensitivity in patients with metabolic syndrome [63,64,65,66]. In contrast, the higher reporting of inflammatory markers with BIS may reflect confounding by indication, as this β1-selective agent is frequently prescribed in patients with chronic obstructive pulmonary disease or renal impairment, conditions themselves associated with systemic inflammation [65,67,68]. Third-generation BBs generally exhibit more favorable metabolic profiles, with CAR improving insulin resistance through its α1-adrenergic vasodilatory effect and NEB enhancing glucose uptake via nitric oxide-mediated mechanisms [63,65,66,69].

A stratified analysis by the HP reporting group was performed to account for potential reporting bias. There are some differences between HPs and non-HPs that could influence the reporting quality: clinical expertise, diagnostic accuracy, and reporting behavior. Thus, performing a stratified disproportionality analysis would yield a more robust assessment of signals. This helps evaluate the consistency of findings across reporting sources.

According to these data, HP reports confirmed higher reporting frequencies of metabolic adverse events for MET. Thus, increases in blood triglycerides and obesity have been reported with MET at 3 times the frequency of BIS, and with T2DM at about 2 times the frequency. Additionally, a significant risk reporting signal for inflammation was observed for MET, with a reporting odds ratio about 3 times higher than for CAR. The results obtained through disproportionality analysis, showing higher reporting odds for metabolic adverse events with MET, could partially be supported by its mechanism, being a second-generation BB, acting selectively on beta-1 receptors. MET might decrease the sensitivity to insulin by reducing blood flow in muscles, the location of glucose intake. Also, lipoprotein lipase inhibition may be triggered by blocking beta receptors, producing an increase in circulating triglyceride. Sensitivity to insulin in the presence of BB was studied by several research groups and the results revealed that MET reduces forearm blood flow during a glucose-plus-insulin infusion [70], but it does not influence insulin sensitivity [70,71]. Regarding triglycerides, the GEMINI trial conducted on diabetic patients showed that in both MET and CAR groups, triglycerides increased, and furthermore, MET increased non-HDL cholesterol [72].

A distinct profile was noted for CAR, which showed higher reporting odds for hyperglycemia and obesity more than 3.5 times as often as BIS. CAR also demonstrated reporting odds for obesity comparable to those observed for MET and the highest reporting odds for hyperglycemia among the evaluated BBs. Although statistically significant, these findings should be interpreted carefully because disproportionality analyses are prone to confounding by indication. Indeed, they contrast with the literature, reporting comparable effects of CAR and MET on clinically relevant hyperglycemia in patients with diabetes [73]. One possible explanation is the preferential prescribing of CAR, especially in patients with diabetes and heart failure [74]. Consequently, greater exposure of high-risk patients to CAR may contribute to selection bias or reverse causality, whereby hyperglycemia reflects the underlying disease rather than an adverse drug interaction.

The above reporting signals detected for MET and CAR were stronger in the HP category (Figure 2b), suggesting that they are present in clinical practice and that physicians are concerned about their impact on patients. Conversely, BIS showed lower reporting odds for metabolic adverse events than MET or CAR. However, NEB showed the lowest reporting odds for metabolic ADRs, which may be influenced by its structure and the smaller number of reports submitted to the EV database. Systematic reviews conducted by independent research groups revealed NEB to have superior vasodilatory and NO-mediated properties [12] compared to other BBs, thus providing a higher benefit to metabolic parameters [17].

Despite a significant number of reports with hepatic steatosis (*n* = 70), no disproportionate reporting signal was identified for any BB agent. This suggests that no beta-blocker-specific reporting signal for hepatic steatosis was identified. Although not many, individual cases of hepatotoxicity to BB were reported [75,76,77], and a general effect was suspected by health authorities [78].

Pharmacovigilance studies complement evidence from clinical trials by identifying rare or long-term adverse events that may not be detected during pre-marketing studies.

Behind the better-known dysglycemic signals, an analysis also highlighted obesity-related outcomes, elevated triglycerides, inflammation-related markers such as increased CRP, and hepatic steatosis as part of the broader metabolic syndrome. These two metabolic adverse effects deserve particular attention because they are less commonly highlighted in routine BB safety discussions. We also showed in this study that hepatic steatosis is not associated with a drug-specific disproportionality signal, but its presence in the dataset suggests that it may be an underappreciated event worth further study.

Our findings support a personalized approach to BB selection, especially in patients with existing cardiometabolic risk factors, such as obesity, T2DM, and dysglycemia. In practical terms, metoprolol and to a lesser extent carvedilol appear to warrant closer monitoring of glucose and lipid parameters, while bisoprolol and especially nebivolol may be preferable when metabolic tolerance is a priority. Clinicians should also monitor weight, triglycerides, and glycemic control after initiation or dose escalation, rather than assuming a uniform class effect. Because the study is based on spontaneous reporting, these recommendations should be framed as risk-aware prescribing and monitoring advice, not as proof of causal harm.

### Limitations

The results of this study should be interpreted within the boundaries of pharmacovigilance database research, which has inherent limitations, first being the lack of a denominator.

Despite data collection being conducted within a clearly established legal and regulatory framework that defines common technical standards for the submission of ICSRs and promotes the use of an internationally harmonized terminology for coding ADRs, spontaneous reporting systems also present several limitations. Probably the two most recognized limitations of these databases remain the underreporting of ADRs and the lack of complete information in the reports. Furthermore, issues such as the misclassification of data, information bias, the continuous expansion of database volume, and variability in safety concerns may also occur. Differences in prescribing frequency and cumulative exposure among the evaluated BBs may have influenced both the number of reports and the disproportionality estimates. As exposure data were not available, it was not possible to adjust for drug utilization; therefore, the observed reporting patterns should not be interpreted as reflecting the true comparative incidence or risk of metabolic adverse events.

The analysis was restricted to four commonly prescribed beta-blockers and did not include other agents, such as atenolol or propranolol. Therefore, the findings should not be generalized to the entire BB class. The relatively small number of reports for several metabolic adverse events precluded meaningful stratified disproportionality analyses according to age, sex, or geographical region.

The quality of reported cases may vary considerably, depending on the level of detail and consistency of the information provided in the reports and on the professional background of the reporter. These limitations were also considered in the interpretation of the results of our study; thus, a sensitivity analysis was performed based on HPs reports. Although causality cannot be established, this type of analysis can help decrease the number of false-positive disproportionality signals, thereby enhancing the overall efficiency of the study. Moreover, disproportionality measures such as the ROR reflect reporting patterns rather than the true incidence or risk of adverse events. Consequently, the identified signals should be considered hypothesis-generating and require confirmation in well-designed epidemiological or clinical studies.

## 4. Materials and Methods

### 4.1. Study Design

This approach included a descriptive and disproportionality analysis on a group of BBs, including four agents (MET, BIS, CAR, and NEB). Metoprolol, bisoprolol, carvedilol, and nebivolol were selected because they are among the most frequently prescribed BBs in contemporary cardiovascular practice. Also, they represent distinct pharmacological profiles within the class, including second- and third-generation agents with different receptor selectivity and vasodilatory properties. This selection enabled a clinically relevant comparison of metabolic ADR reporting among commonly used BBs.

Individual Case Safety Reports (ICSRs) submitted to the EudraVigilance (EV) portal www.adrreports.eu (accessed on 28 and 29 April 2026) database [79] up until 26 April 2026 were assessed. Data were collected between 28 and 29 April 2026. According to the Medical Dictionary for Regulatory Activities (MedDRA), different classification levels are used to report potential ADRs [80]. Thus, each ADR is reported under a preferred term (PT) within a System Organ Class (SOC), which represents the highest level of the hierarchy. PT is a distinct and unique medical concept, the most frequently used for reporting symptoms, diagnostics, investigations, or procedures, whereas SOC groups terms by physiologic and pathophysiologic criteria [81]. The reporting of metabolic ADRs associated with BB therapy was investigated (dyslipidemia, hyperglycemia, and type 2 diabetes). For analytical purposes, 20 PTs were grouped into categories (Table 4). The selected PTs were identified a priori based on published evidence regarding BB-associated metabolic ADRs and their clinical relevance and were subsequently grouped into predefined pathophysiological categories.

Approval from an ethics committee was not required because this study utilizes aggregated ICSRs from which all personal identifiers have been removed, ensuring patient confidentiality [82].

### 4.2. Descriptive and Disproportionality Analysis

Descriptive analysis of the general characteristics, based on aggregated data retrieved from the EV portal (age category, sex, report origin, and reported category), was performed [83]. The EV public dashboard provides aggregated data after routine data management by the EMA; therefore, duplicate reports could not be independently identified or removed. The frequencies of reports by SOC and PTs of interest were also analyzed [84,85]. Furthermore, signal detection at the PT level was followed by the distribution of reports for selected metabolic ADRs associated with the evaluated BBs. A disproportionality analysis was performed to compare the reporting odds of selected metabolic adverse events among the evaluated BBs in the overall dataset and in the HP-restricted dataset. Stratifying the disproportionality analysis using HP reports was performed to provide a more robust assessment of the metabolic signals for MET, BIS, CAR, and NEB.

Each drug was compared with the remaining BBs included in the analysis.

The reporting odds ratio (ROR) with a 95% confidence interval (CI) was used for signal detection, as it is the EMA-recommended method for the EV database. RORs were calculated from standard 2 × 2 contingency tables using the presence or absence of the selected PT for the index drug versus the comparator drugs.

A signal was considered statistically significant when the lower limit of the 95% CI exceeded 1.

Exclusion criteria: BB fixed-drug combinations were excluded from this study. Also, some PTs were excluded from the disproportionality analysis. Thus, of the twenty selected PTs, three reported no ADRs. For the remaining PTs, the disproportionality analysis could not be performed for nine PTs due to the low number of reports (n < 5).

## 5. Conclusions

Beta-blockers remain an essential therapy for cardiovascular diseases; however, important reporting differences were observed in this large pharmacovigilance analysis on the European database EV. As medication needs to be both effective and safe, this study investigated the reporting patterns of metabolic disturbances for some of the commonly prescribed BBs in outpatient treatment. Our findings indicate that metoprolol and carvedilol are associated with significantly higher reporting odds of glycemic and lipid disturbances than other BBs. On the other hand, bisoprolol and especially nebivolol showed lower reporting in this category. These events, validated through high-quality professional reports, suggest a need for individualized therapy and enhanced metabolic monitoring in patients prescribed these specific agents, particularly those with pre-existing risk factors. The results should be interpreted taking into consideration the limitations generated from the spontaneous reporting system, which does not permit the establishment of causal relationships.

## Figures and Tables

**Table 1:** General characteristics of ICSRs reported in EudraVigilance for beta-blocker drugs.

	Metoprolol	Carvedilol	Bisoprolol	Nebivolol
	*n* (%)	*n* (%)	*n* (%)	*n* (%)
Age				
0–1 Months	141	10	124	20
	(0.50%)	(0.10%)	(0.60%)	(0.40%)
2 Months–2 Years	54	38	46	10
	(0.20%)	(0.40%)	(0.20%)	(0.20%)
3–11 Years	51	29	57	11
	(0.20%)	(0.30%)	(0.30%)	(0.20%)
12–17 Years	169	43	77	25
	(0.60%)	(0.50%)	(0.40%)	(0.50%)
18–64 Years	10,312	2830	5715	1794
	(38.00%)	(29.80%)	(26.10%)	(34.30%)
65–85 Years	9631	3922	9520	2124
	(35%)	(41.30%)	(43.50%)	(40.60%)
More than 85 Years	1785	760	2659	439
	(6.60%)	(8.00%)	(12.20%)	(8.40%)
NS	5026	1855	3662	805
	(18.50%)	(19.60%)	(16.80%)	(15.40%)
Sex				
Female	14,600	3981	11,469	2869
	(53.70%)	(42.00%)	(52.50%)	(54.90%)
Male	11,720	5049	9868	2250
	(43.10%)	(53.20%)	(45.10%)	(43.00%)
NS	849	457	523	109
	(3.10%)	(4.80%)	(2.40%)	(2.10%)
Origin				
EEA	11,638	3144	17,028	4197
	(42.80%)	(33.10%)	(77.90%)	(80.30%)
Non-EEA	15,529	6343	4832	1031
	(57.20%)	(66.90%)	(22.10%)	(19.70%)
NS	2	0	0	0
	(0.00%)	(0.00%)	(0.00%)	(0.00%)
Reporter category				
HPs	18,835	7945	16,379	3879
	(69.30%)	(83.70%)	(74.90%)	(74.20%)
Non-HPs	8203	1458	5396	1342
	(30.20%)	(15.40%)	(24.70%)	(25.70%)
NS	131	84	85	7
	(0.50%)	(0.90%)	(0.40%)	(0.10%)

**Table 2:** Frequency of ADRs by SOCs.

	Frequency				Rank			
	Metoprolol	Carvedilol	Bisoprolol	Nebivolol	Metoprolol	Carvedilol	Bisoprolol	Nebivolol
Blood and lymphatic system disorders	2.8%	5.0%	3.1%	2.5%	rank: 19	rank: 15	rank: 17	rank: 17
Cardiac disorders	30.1%	32.0%	28.8%	21.2%	rank: 2	rank: 1	rank: 1	rank: 3
Congenital, familial and genetic disorders	0.8%	0.3%	0.5%	0.5%	rank: 27	rank: 27	rank: 27	rank: 25
Ear and labyrinth disorders	2.6%	1.5%	3.2%	3.3%	rank: 20	rank: 21	rank: 16	rank: 15
Endocrine disorders	1.0%	0.9%	0.6%	0.7%	rank: 25	rank: 24	rank: 25	rank: 24
Eye disorders	4.9%	3.9%	3.9%	4.8%	rank: 15	rank: 16	rank: 15	rank: 14
Gastrointestinal disorders	15.7%	12.3%	14.1%	14.5%	rank: 8	rank: 8	rank: 8	rank: 7
General disorders and administration site conditions	32.6%	31.2%	27.4%	28.5%	rank: 1	rank: 2	rank: 2	rank: 1
Hepatobiliary disorders	2.2%	3.8%	2.4%	1.8%	rank: 21	rank: 17	rank: 19	rank: 20
Immune system disorders	2.9%	1.9%	1.8%	1.5%	rank: 18	rank: 20	rank: 21	rank: 21
Infections and infestations	5.7%	5.9%	4.4%	2.9%	rank: 13	rank: 14	rank: 14	rank: 16
Injury, poisoning and procedural complications	23.8%	14.9%	15.8%	14.9%	rank: 4	rank: 7	rank: 5	rank: 5
Investigations	20.2%	19.5%	15.7%	14.7%	rank: 5	rank: 4	rank: 6	rank: 6
Metabolism and nutrition disorders	7.5%	11.0%	7.3%	6.5%	rank: 12	rank: 9	rank: 12	rank: 12
Musculoskeletal and connective tissue disorders	9.2%	6.1%	7.5%	7.5%	rank: 11	rank: 13	rank: 11	rank: 11
Neoplasms benign, malignant and unspecified (incl. cysts and polyps)	3.1%	2.0%	1.3%	1.5%	rank: 16	rank: 19	rank: 22	rank: 22
Nervous system disorders	26.1%	23.5%	23.8%	23.9%	rank: 3	rank: 3	rank: 3	rank: 2
Pregnancy, puerperium and perinatal conditions	1.8%	0.7%	1.1%	0.5%	rank: 23	rank: 26	rank: 23	rank: 26
Product issues	3.0%	1.2%	2.6%	2.0%	rank: 17	rank: 23	rank: 18	rank: 19
Psychiatric disorders	19.1%	10.3%	10.3%	10.4%	rank: 6	rank: 10	rank: 10	rank: 10
Renal and urinary disorders	5.0%	8.3%	5.9%	5.5%	rank: 14	rank: 12	rank: 13	rank: 13
Reproductive system and breast disorders	2.0%	1.4%	1.9%	2.3%	rank: 22	rank: 22	rank: 20	rank: 18
Respiratory, thoracic and mediastinal disorders	14.4%	15.8%	12.9%	12.4%	rank: 9	rank: 6	rank: 9	rank: 9
Skin and subcutaneous tissue disorders	11.5%	9.1%	14.3%	14.5%	rank: 10	rank: 11	rank: 7	rank: 7
Social circumstances	1.0%	0.8%	0.5%	0.4%	rank: 26	rank: 25	rank: 26	rank: 27
Surgical and medical procedures	1.5%	3.3%	1.0%	0.8%	rank: 24	rank: 18	rank: 24	rank: 23
Vascular disorders	17.2%	17.1%	16.2%	15.8%	rank: 7	rank: 5	rank: 4	rank: 4

**Table 3:** Distribution of cases related to selected metabolic disorders reported for beta-blockers.

SOC	Pathophysiological Category	PT	MET	CAR	BIS	NEB	Total
Metabolism and nutrition disorders	Blood glucose disorders	Hyperglycamia	73	49	30	3	155
Metabolism and nutrition disorders	Blood glucose disorders	Type 2 diabetes mellitus	99	22	28	6	155
Investigations	Inflammation	C-reactive protein increased	25	30	62	1	118
General disorders and administration site conditions	Inflammation	Inflammation	58	8	38	6	110
Investigations	Lipid disorders	Blood triglycerides increased	64	7	28	4	103
Hepatobiliary disorders	Hepatic disorders	Hepatic steatosis	36	9	17	8	70
Metabolism and nutrition disorders	Obesity	Obesity	37	13	11	2	63
Investigations	Lipid disorders	High-density lipoprotein decreased	21	1	14	0	36
Metabolism and nutrition disorders	Blood glucose disorders	Glucose tolerance impaired	16	7	6	0	29
Investigations	Inflammation	Inflammatory marker increased	2	0	11	3	16
Investigations	Obesity	Waist circumference increased	4	2	7	0	13
Metabolism and nutrition disorders	Metabolic syndrome	Metabolic syndrome	8	1	3	1	13
General disorders and administration site conditions	Inflammation	Systemic inflammatory response syndrome	7	3	2	0	12
Metabolism and nutrition disorders	Blood glucose disorders	Impaired fasting glucose	3	0	1	0	4
Metabolism and nutrition disorders	Blood glucose disorders	Insulin resistance	1	1	1	1	4
Metabolism and nutrition disorders	Obesity	Central obesity	0	1	0	2	3
Investigations	Inflammation	Interleukin level increased	1	1	0	0	2
Investigations	Inflammation	Alpha tumor necrosis factor increased	0	0	0	0	0
Metabolism and nutrition disorders	Blood glucose disorders	Diabetes mellitus non-insulin-dependent	0	0	0	0	0
Metabolism and nutrition disorders	Blood glucose disorders	Hyperglycemic unconsciousness	0	0	0	0	0

**Table 4:** Mapping of PTs to pathophysiological categories.

Pathophysiological Category	SOC	PT
Dysglycemia and Type 2 Diabetes Spectrum	Metabolism and nutrition disorders	hyperglycemia
Dysglycemia and Type 2 Diabetes Spectrum	Metabolism and nutrition disorders	type 2 diabetes mellitus
Dysglycemia and Type 2 Diabetes Spectrum	Metabolism and nutrition disorders	glucose tolerance impaired
Dysglycemia and Type 2 Diabetes Spectrum	Metabolism and nutrition disorders	impaired fasting glucose
Dysglycemia and Type 2 Diabetes Spectrum	Metabolism and nutrition disorders	insulin resistance
Dysglycemia and Type 2 Diabetes Spectrum	Metabolism and nutrition disorders	diabetes mellitus non-insulin-dependent
Dysglycemia and Type 2 Diabetes Spectrum	Metabolism and nutrition disorders	hyperglycemic unconsciousness
Inflammatory Disorders and Biomarkers	Investigations	C-reactive protein increased
Inflammatory Disorders and Biomarkers	General disorders and administration site conditions	inflammation
Inflammatory Disorders and Biomarkers	Investigations	inflammatory marker increased
Inflammatory Disorders and Biomarkers	General disorders and administration site conditions	systemic inflammatory response syndrome
Inflammatory Disorders and Biomarkers	Investigations	interleukin level increased
Inflammatory Disorders and Biomarkers	Investigations	alpha tumor necrosis factor increased
Metabolic Syndrome and Obesity-Related Disorders	Hepatobiliary disorders	hepatic steatosis
Metabolic Syndrome and Obesity-Related Disorders	Investigations	blood triglycerides increased
Metabolic Syndrome and Obesity-Related Disorders	Investigations	high-density lipoprotein decreased
Metabolic Syndrome and Obesity-Related Disorders	Metabolism and nutrition disorders	metabolic syndrome
Metabolic Syndrome and Obesity-Related Disorders	Metabolism and nutrition disorders	obesity
Metabolic Syndrome and Obesity-Related Disorders	Investigations	waist circumference increased
Metabolic Syndrome and Obesity-Related Disorders	Metabolism and nutrition disorders	central obesity

## Data Availability

The original contributions presented in this study are included in the article. Further inquiries can be directed to the corresponding author.
